# Metabolic Stability of D-Allulose in Biorelevant Media and Hepatocytes: Comparison with Fructose and Erythritol

**DOI:** 10.3390/foods8100448

**Published:** 2019-10-01

**Authors:** Han-Joo Maeng, Jin-Ha Yoon, Kwang-Hoon Chun, Sung Tae Kim, Dong-Jin Jang, Ji-Eun Park, Yang Hee Kim, Seong-Bo Kim, Yu Chul Kim

**Affiliations:** 1 College of Pharmacy, Gachon University, Incheon 21936, Korea; hjmaeng@gachon.ac.kr (H.-J.M.); jinha89@daum.net (J.-H.Y.); khchun@gachon.ac.kr (K.-H.C.); 2 Department of Pharmaceutical Engineering, Inje University, Gimhae 50834, Korea; stkim@inje.ac.kr (S.T.K.); djjang@inje.ac.kr (D.-J.J.); 3 Food Research Institute, CJ CheilJedang Corp., Suwon 16495, Korea; je.park12@cj.net (J.-E.P.); yanghee.kim@cj.net (Y.H.K.)

**Keywords:** D-allulose, D-fructose, erythritol, metabolic stability, biorelevant media, hepatocytes

## Abstract

D-allulose, a C-3 epimer of D-fructose, is a rare monosaccharide used as a food ingredient or a sweetener. In the present study, the in vitro metabolic stability of D-allulose was examined in biorelevant media, that is, simulated gastric fluid (SGF) and fasted state simulated intestinal fluid (FaSSIF) containing digestive enzymes, and in cryopreserved human and rat hepatocytes. The hepatocyte metabolic stabilities of D-allulose were also investigated and compared with those of fructose and erythritol (a sugar-alcohol with no calorific value). D-allulose was highly stable in SGF (97.8% remained after 60 min) and in FaSSIF (101.3% remained after 240 min), indicating it is neither pH-labile nor degraded in the gastrointestinal tract. D-allulose also exhibited high levels of stability in human and rat hepatocytes (94.5–96.8% remained after 240 min), whereas fructose was rapidly metabolized (43.1–52.6% remained), which suggested these two epimers are metabolized in completely different ways in the liver. The effects of D-allulose on glucose and fructose levels were negligible in hepatocytes. Erythritol was stable in human and rat hepatocytes (102.1–102.9% remained after 240 min). Intravenous pharmacokinetic studies in rats showed D-allulose was eliminated with a mean half-life of 72.2 min and a systemic clearance of 15.8 mL/min/kg. Taken together, our results indicate that D-allulose is not metabolized in the liver, and thus, unlikely to contribute to hepatic energy production.

## 1. Introduction

Obesity is defined as abnormal or excessive fat accumulation and has been proven to be a serious health risk [1,2]. The prevalence of obesity has dramatically increased over the past decades in developing and developed countries, and thus, obesity presents a serious worldwide public health problem [1]. Furthermore, chronic obesity is a major risk factor of serious metabolic and cardiovascular diseases including type 2 diabetes, dyslipidemia, hypertension, coronary heart disease, and stroke [1,2]. Various causes of obesity have been suggested and identified causative agents include dietary components [3]. In particular, diets containing simple sugars like sucrose and high-fructose corn syrup (HFCS; as a fructose source) have been associated with excessive caloric intake [3]. In addition, it has been reported that the risk of metabolic disease is increased by the consumption of fructose-containing sugar via the direct and indirect pathways [4], and similarly, glucose is known to have cardiometabolic effects [5]. Therefore, several low or no-calorie sweeteners have been developed for use as sugar substitutes in foods and beverages to combat the increased prevalence of obesity and related comorbidities.

**Figure 1:**
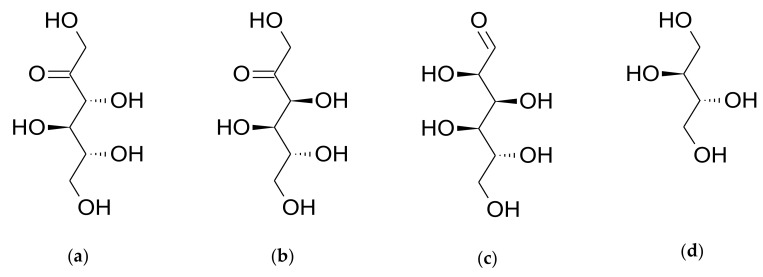
Chemical structures of (**a**) D-allulose, (**b**) D-fructose, (**c**) D-glucose, and (**d**) erythritol.

D-allulose (also known as D-psicose, Figure 1a) is a C-3 epimer of fructose and is present in only limited quantities in nature. D-allulose has 70% of the sweetness of sucrose, which means it is viewed as a potential alternative sweetener [6,7]. Interestingly, D-allulose does not increase blood sugar levels, has minimal calorific value in vivo [8,9], and has been shown to have beneficial physiological effects, which include improved glucose tolerance, increased insulin sensitivity, and reduced body weight gain [10,11,12,13,14].

The US Food and Drug Administration (FDA) has designated D-allulose as ‘generally regarded as safe’ (GRAS) for use as a food ingredient, but initially stipulated that amounts of D-allulose added be included in total and added sugar contents on product nutritional labels. However, in response to recent scientific evidence [15,16], the FDA changed this stipulation and decided D-allulose could be excluded from total and added sugar contents on product labels [17], and that for labeling purposes a caloric value of 0.4 kcal/g (instead of 4 kcal/g) should be used to estimate the caloric contribution of D-allulose. Recent studies have shown D-allulose is rapidly eliminated via the urinary route and has negligible calorific value in humans or rats. Tissue distribution studies have shown D-allulose is mainly distributed to liver [18]. Several in vivo pharmacokinetic studies have been conducted on the metabolism and excretion of D-allulose [16,18,19], but its in vitro metabolism by hepatocytes, which are largely responsible for metabolizing caloric sugars, has not been previously reported.

The liver is responsible for controlling the metabolism of galactose, fructose, glucose, and other carbohydrates, and glucose homeostasis is maintained by the regulation of transporters and enzymes expressed in hepatocytes. Dietary carbohydrates are readily absorbed in the intestines and pass through the liver via the portal vein before reaching the systemic circulation. Primary hepatocytes are viewed as a physiologically relevant system for the evaluation of nutrient metabolism, and thus, have been used in metabolic studies of drugs and nutrient (e.g., lipids and carbohydrates) [20,21,22]. Furthermore, cryopreserved hepatocytes held in suspension have been recommended for use in short-term metabolic and toxicity studies [22], and reports have consistently shown good correlations between results obtained in vitro using hepatocytes and in vivo derived data [23]. However, the metabolism of rare sugars and sugar alcohols have not been studied using hepatocytes. In the present study, the in vitro and in vivo pharmacokinetics of D-allulose were investigated in human and rat hepatocytes and in Sprague-Dawley rats to determine whether D-allulose is involved in energy production via hepatic metabolism. In addition, we compared the metabolic stabilities of fructose and erythritol (a caloric monosaccharide and non-caloric sugar alcohol, respectively) with that of D-allulose, and evaluated the stabilities of D-allulose at absorption sites using bio-relevant media, namely, simulated gastric fluid (SGF) and fasted state simulated intestinal fluid (FaSSIF).

## 2. Materials and Methods 

### 2.1. Chemicals and Reagents

The chemical structures of the four test compounds are presented in Figure 1 and their physico-chemical characteristics are presented in Table 1. D-Allulose (Figure 1a), D-fructose (Figure 1b), D-glucose (Figure 1c), and erythritol (Figure 1d) were all supplied by CJ CheilJedang Corp. (Suwon, South Korea). Pooled rat and human cryopreserved hepatocytes were purchased from XenoTech (Kansas City, KS, USA). FaSSIF/FeSSIF/FaSSGF powder (product code: FFF01) was obtained from Biorelevent.com (London, UK). Pancreatin and pepsin were purchased from TCI chemicals (Tokyo, Japan) and Sigma-Aldrich (St. Louis, MO, USA), respectively. Salicin (the internal standard (IS) used for liquid chromatography-tandem mass spectrometry (LC-MS/MS) analysis) was purchased from Sigma-Aldrich (St. Louis, MO, USA). Water was purified using the aquaMAX™, ultra-pure water purification system (YL Instruments, Anyang, Korea). All other chemicals and solvents were of reagent or high-performance liquid chromatography (HPLC) grade and used without further purification.

### 2.2. Preparation of Assay Media for The Stability Study

#### 2.2.1. FaSSIF and SGF

The simulated gastrointestinal fluids were prepared using FaSSIF/FeSSIF/FaSSGF powder (www.biorelevant.com), according to manufacturer’s instructions. For blank SGF buffer, 2.0 g of sodium chloride was dissolved in 900 mL of purified water, adjusting pH to 1.6 with hydrochloric acid, and diluting to a final volume of 1000 mL For blank FaSSIF buffer, 0.105 g of sodium hydroxide pellets, 0.8510 g of monobasic sodium phosphate (anhydrous), and 1.547 g of sodium chloride were dissolved in 230 mL of water, and after adjusting the pH to 6.5 with sodium hydroxide or hydrochloric acid, and then the buffer was made by diluting to 250 mL with purified water.

SGF and FaSSIF solutions were prepared fresh on days of experiments using FaSSIF/FeSSIF/FaSSGF powder. SGF solution (final volume 1000 mL) was prepared by adding 0.0597 g of FaSSIF/FeSSIF/FaSSGF powder and 3.2 g of pepsin (from porcine gastric mucosa) to blank SGF buffer. FaSSIF/FeSSIF/FaSSGF powder (0.560 g) and 2.5 g of pancreatin (from porcine pancreas) was added to blank FaSSIF buffer to make FaSSIF solution (final volume 250 mL).

#### 2.2.2. Hepatocytes

Cryopreserved rat (R1000.H15; male IGS SD rat) and human (CryostaX HPCH10; Pooled from 10 donors; five males and five females, overall age range 7–67 years, one African American and 9 Caucasians) hepatocytes were thawed at 37 °C, transferred to OptiThaw medium (XenoTech), centrifuged at 100× *g* for 5 min, and resuspended in incubation medium (OptiIncubate, XenoTech). Cell viabilities were determined by Trypan blue exclusion using the OptiThaw Hepatocyte Kit (Xenotech). Finally, hepatocytes were diluted to 2.0 × 10^6^ cells/mL in OptiIncubate medium for the stability assay, which was conducted according to the manufacture’s instruction.

### 2.3. Preliminary Stability Assay

The stability of D-allulose was initially investigated in phosphate-buffered saline (PBS) to check its non-specific degradation under normal assay condition at pH 7.4. Briefly, D-allulose was incubated in PBS at 500 μg/mL for 240 min at 37 °C. Incubates were sampled at 0, 30, and 240 min for the LC-MS/MS analysis of D-allulose. Fructose and glucose levels were also monitored throughout the incubation period to determine whether D-allulose was converted to other monosaccharides under assay conditions.

### 2.4. Metabolic Stability in Simulated Gastrointestinal Fluids

The metabolic stability of D-allulose was investigated in SGF and in FaSSIF. A stock solution of D-allulose was prepared in purified water and diluted to a final concentration of 500 μg/mL in incubation medium. The final incubation volume was 500 μL. To terminate reactions, 50 μL aliquots of incubates were collected after 0, 60 and 120 min of incubation for SGF or at 0, 60, 120, and 240 min for FaSSIF. The aliquots were added to 200 μL of acetonitrile (ACN) containing IS (salicin, 25 μg/mL) (as was previously described for LC-MS/MS assay of carbohydrates including fructose and glucose in plasma [24]) and centrifuged for 5 min at 15,000 rpm to eliminate pepsin or pancreatin aggregates. Supernatants were stored at −70 °C prior to analysis.

### 2.5. Metabolic Stability in Primary Hepatocytes

Metabolic stability assays for D-allulose, fructose, or erythritol in human and rat hepatocytes were performed as previously described [25] with minor modification. Stock solutions of test substance were prepared in purified water and diluted to 1 mg/mL in OptiIncubate Hepatocyte Media (K8400). Solutions (0.25 mL) were then placed in the wells of 24-well non-coated plates and preincubated at 37 °C. Incubations were initiated by adding 0.25 mL of cell suspension to the wells to a final volume of 0.5 mL (final test substance concentration 500 μg/mL and final cell concentration 1 × 10^6^ cells/mL). Reactions were conducted in a humidified incubator with cells dispersed using an orbital shaker at 120 rpm. Incubations were terminated 0, 30, 60, 120, and 240 min later by transferring 50 μL aliquots from wells into tubes containing 200 μL of ACN including IS (salicin, 25 μg/mL). Samples were centrifuged for 5 min at 15,000 rpm and supernatants were stored frozen at −70 °C prior to analysis. Since incubation media contained 11 mM of glucose to maintain hepatocyte viability, negative controls were prepared using incubation media containing 11 mM of glucose and were run in parallel using the same sampling times as other groups.

### 2.6. In Vivo Systemic Pharmacokinetic Study in Rats

Pharmacokinetic studies on D-allulose were performed in male Sprague-Dawley rats (7–8 weeks old, 220–280 g) obtained from Orient Bio Inc. (Seongnam, Korea). Rats were maintained under a standard 12:12 h light/dark cycle for 1 week. All experiments complied with the Guidelines for Animal Care and Use issued by Gachon University. Experimental animal protocols were reviewed and approved by the Animal Care and Use Committee of the Gachon University (#GIACUC-R2018022, approval date 7 November 2018). As described in our recent pharmacokinetic study [26], overnight fasted rats were anesthetized intramuscularly with Zoletil (20 mg/kg), and a femoral vein and artery were then surgically cannulated with polyethylene tubing (PE50; Clay Adams, Parsippany, NJ, USA). The cannula was flushed with 20 IU/mL heparinized saline to prevent clotting. After rats had recovered from anesthesia, D-allulose solution in saline (50 mg/ mL) was injected intravenously at 100 mg/kg (*n* = 5). Blood samples (100 μL) were collected from a femoral artery at 2, 10, 30, 60, 120, 240, and 480 min after D-allulose administration, immediately centrifuged at 14,000 rpm for 15 min at 4 °C, and the plasma obtained was stored at −70 °C prior to LC-MS/MS analysis.

### 2.7. LC-MS/MS Analysis

LC-MS/MS was performed in negative electrospray ionization (ESI^-^) mode on a tandem quadrupole mass spectrometer (Agilent Technologies) with an electrospray interface. Multiple reaction monitoring (MRM) was selected to quantify test substances and the IS using a slight modification of a previously described method [24]. In detail, the LC-MS/MS system consisted of an Agilent HPLC system (1290 Infinity, Agilent Technologies, Santa Clara, CA, USA) and an Agilent 6490 QQQ mass spectrometer with an ESI^-^ Agilent Jet Stream ion source (Agilent Technologies, Santa Clara, CA, USA). Carbohydrates were separated using a Luna amino column (150 mm × 2 mm ID, 3 μm particle size; Phenomenex, Torrance, CA, USA) at a flow rate of 0.2 mL/min. The mobile phase consisted of ACN and water (80:20). Analytical data was acquired using Mass Hunter software (version A.02.00; Agilent Technologies, Santa Clara, CA, USA). Concentrations of glucose and fructose were determined before and after incubating D-allulose in PBS, SGF, FaSSIF, and hepatocytes.

### 2.8. Data Analysis

In the metabolic study, the area ratios (test compound/IS) as determined by LC-MS/MS were used to calculate percentages (%) of the test substances remaining at different time points versus time zero. Percentages remaining were plotted versus time and in vitro hepatic intrinsic clearances (CL_int, in vitro_) were calculated using Equation (1).

CL_int, in vitro_ = 0.693/t_1/2_ (min)/hepatocyte concentration (10^6^ cells/mL) (where t_1/2_ = elimination half-life) (1)

CL_int, in vitro_ values were scaled to in vivo intrinsic clearance (CL_int, in vivo_) using physiological parameters (Table 2) as follows (Equation (2)) [27,28].

CL_int, in vivo_ = CL_int, in vitro_ × Number of hepatocyte (cells/g liver) × liver weight (g liver/kg body weight) (2)

Finally, predicted hepatic clearances (CL_h, in vivo_) were calculated using the ‘well-stirred’ liver clearance model [29] and hepatic extraction ratio (E_h_) were calculated as follows (Equations (3) and (4)). Unbound fractions of test substances in blood (f_u,b_) were assumed to be 1 when CL_h, in vivo_ and E_h_ were calculated.

CL_h, in vivo_ = (Q_h_ × f_u,b_ × CL_int, in vivo_)/(Q_h_ + f_u,b_ × CL_int, in vivo_) (3)

E_h_ = f_u,b_ × CL_int, in vivo_/(Q_h_ + f_u,b_ × CL_int, in vivo_) (Where Q_h_ = Hepatic blood flow, f_u,b_ = unbound fraction in blood, assumed as 1) (4)

Noncompartmental analysis was performed using WinNonlin program (Ver. 5.0.1., Pharsight Corporation, Mountain View, CA, USA)to calculate pharmacokinetic parameters, that is, area under the plasma concentration-time curve (AUC), systemic clearance (CL), elimination half-life (t_1/2_), volume of distribution at steady state (V_ss_) and mean residence time (MRT).

### 2.9. Statistical Analysis

One-way ANOVA (Analysis of Variance) with Tukey’s post-hoc test was used to compare percentages of test substances remaining in hepatocytes after incubation for 240 min. The significances of changes in the concentrations of glucose and fructose after incubating hepatocytes with D-allulose were analyzed using the paired *t*-test. Statistical significance was accepted for *p* values < 0.05.

## 3. Results and Discussion

### 3.1. Analytical Methods Using LC-MS/MS

We first optimized MS conditions for collision energy and cell accelerator voltage with respect to MRM transition, for D-allulose, fructose, glucose, erythritol, and salicin (IS). Details of the optimized ESI-MS parameters including MRM conditions are summarized in Table 3.

Using optimized LC-MS/MS conditions, calibration curves for D-allulose, fructose, glucose, and erythritol were linear with correlation coefficients (r) of > 0.990 as determined by weighted (1/x^2^) least-square regression analysis, which showed LC-MS/MS response was proportional to carbohydrates concentrations.

### 3.2. Metabolic Stability Study

#### 3.2.1. Stability of D-allulose in PBS (pH 7.4)

**Figure 2:**
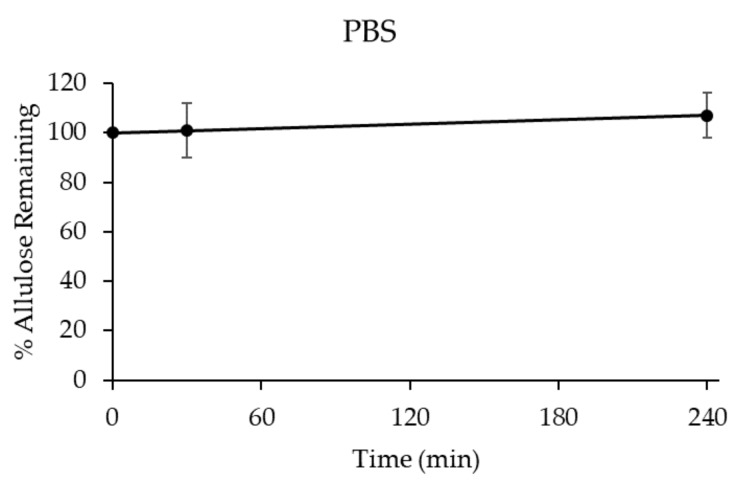
Percentages of D-allulose remaining as a function of time after incubation in PBS. Results are presented as means and error bars represent standard deviations. (*n* = 5 at each time point).

D-allulose (500 μg/mL) was incubated in PBS and D-allulose levels were determined after incubation for 0, 30, and 240 min. Percentages of D-allulose remaining were calculated as described in Materials and Methods. Glucose and fructose levels were also determined before and after incubation. D-allulose was stable for up to 240 min in PBS (Figure 2), but glucose and fructose were not detected. These results suggest that D-allulose is not degraded under physiological or general assay condition.

#### 3.2.2. Stability of D-allulose in Biorelevant Media

**Figure 3:**
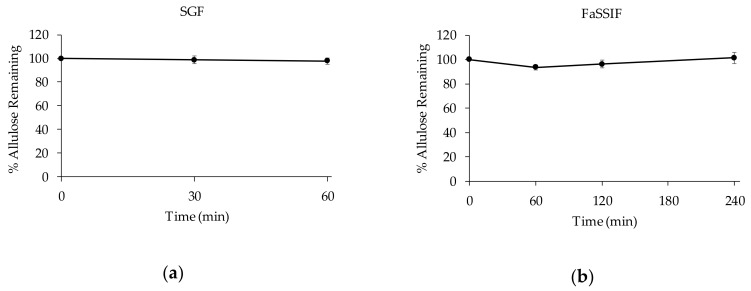
Percentages of D-allulose remaining as a function of time following incubation in (**a**) simulated gastric fluid (SGF) or (**b**) fasted state simulated intestinal fluid (FaSSIF). Results are presented as means and error bars represent standard deviation. (*n* = 5 at each time point).

When ingested as a food additive, D-allulose passes through the gastrointestinal tract and while in transit encounters various pH conditions and digestive enzymes (e.g., pepsin and pancreatin). To determine whether D-allulose is stable in gastrointestinal fluids, its stability was examined in simulated gastric fluid (SGF) containing pepsin and in fasted state simulated intestinal fluid (FaSSIF) containing pancreatin. D-allulose (500 μg/mL) was incubated in SGF or FaSSIF for 60 or 240 min, respectively, and remaining percentages of D-allulose were calculated. D-allulose proved to be stable in SGF or FaSSIF for 60 and 240 min, respectively, which indicated it is not sensitive to pH or to enzymatic degradation in the gastrointestinal tract (Figure 3).

Neither glucose nor fructose was detected after incubating D-allulose in SGF. Fructose was not detected in FaSSIF, but glucose was detected in FaSSIF at baseline and after 4 h at 25.7 and 52.7 μg/mL, respectively. Since D-allulose was highly stable in FaSSIF and glucose was detected before incubation, this result was probably due to the constituents of pancreatin obtained from porcine pancreas. Sucrose is commonly added to pancreatin products as an enzyme activity stabilizer [30]. Actually, pancreatin solutions were found to contain considerable amounts of glucose in the present study (data not shown).

#### 3.2.3. Stability of D-allulose, Glucose, Fructose, and Erythritol in Hepatocytes

**Figure 4:**
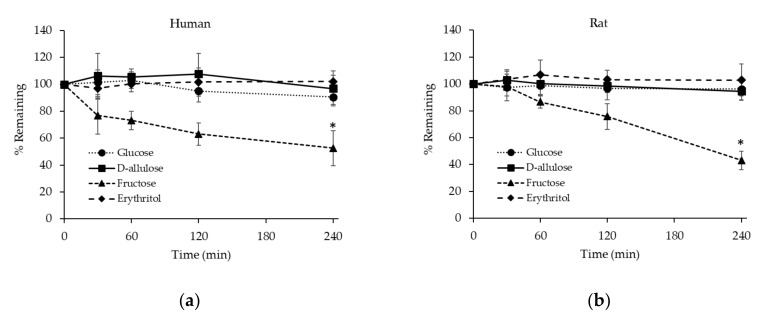
Percentages of test substances remaining as a function of time after incubation in human (**a**) or rat (**b**) hepatocytes. Results are presented as means and error bars represent standard deviations. (*n* = 5 at each time point). ^*^ Significantly different from the other groups (*p* < 0.001).

Because the incubation medium used in the hepatocyte metabolic stability assays contained 11 mM of glucose, the stability of glucose in hepatocytes was examined without adding extra glucose. Human and rat hepatocytes were incubated in the medium and glucose levels were determined after incubation for 30, 60, 120, and 240 min. Glucose levels were stable for up to 240 min in human and rat hepatocytes (Figure 4). Similarly, Bissel et al. reported a very low rate of glucose utilization by hepatocytes in primary culture over 24 h [31].

The metabolic stability of D-allulose was also examined in human and rat hepatocytes. D-allulose (500 μg/mL) was incubated in human or rat hepatocytes and its concentrations were determined after incubation for 30, 60, 120, and 240 min. D-allulose proved to be stable for up to 240 min in human and rat hepatocytes indicating negligible metabolism (Figure 4). Glucose and fructose levels were also determined before and after incubating D-allulose in hepatocytes to examine the effect of D-allulose on their metabolism. As was observed for glucose-only incubation, glucose levels were not significantly changed after incubation with D-allulose for 240 min (97.1 and 95.4% remained after 240 min in human and rat hepatocytes, respectively). Fructose was detected at low levels before and after incubating D-allulose in human or rat hepatocytes, but its concentration was not significantly changed by either incubation (for human hepatocytes before and after incubation 1.26 ± 0.19 versus 1.24 ± 0.41 μg/mL, respectively, and for rat hepatocytes 1.04 ± 0.33 versus 0.97 ± 0.27 μg/mL). These results show D-allulose is barely metabolized by hepatocytes and that it has negligible effect on the metabolism of glucose or fructose by hepatocytes.

The metabolic stabilities of fructose and erythritol were evaluated in the same manner as that described for D-allulose. Percentages of fructose remaining markedly decreased after incubation for 240 min in human or rat hepatocytes, suggesting rapid metabolism of fructose in liver (Figure 4), and that the enzymes involved in fructolysis in human or rat liver are substrate-specific. Consistently, liver has been reported as the organ that predominantly extracts fructose, probably because of the high affinity and insensitivity to cellular energy status of fructokinase in liver [32]. Indeed, isotopic tracer studies have shown that fructose is substantially converted into glucose when co-ingested with glucose in vivo: Fructose was metabolized into glucose (28.9–54%), lactate (~28%), glycogen (17%), and triacylglycerol (<1%) in <6 h [33,34]. Erythritol was included as a reference compound because it is a known zero-calorie sugar alcohol [35]. As was expected, erythritol was stable for up to 240 min in human or rat hepatocytes, indicating negligible metabolism in liver (Figure 4).

CL_int, in vitro_ could be calculated only for fructose because the other test compounds were highly stable in hepatocytes after 240 min of incubation (Table 4).

Dietary sources of fructose include fruit, high fructose corn syrup, and fructose as a component of sucrose. Studies suggest that high fructose intake is associated with the onset of obesity, and thus, with various metabolic and cardiovascular diseases [3,4]. Even though glucose and fructose have the same caloric values, they are metabolized differently in the body [36,37]. Briefly, glucose is circulated in blood and utilized as energy source by most cells. Glycolysis, the metabolic pathway that converts glucose to pyruvate, is regulated by several enzymes, especially phosphofructokinase-1 (PFK-1). In an energy-depleted state, PFK-1 is positively regulated by insulin, fructose 2,6-bisphosphate, and AMP, and in a state of energy surplus, it is negatively regulated by glucagon, ATP, and citrate. Unlike glucose, fructose is almost entirely taken up by liver and metabolized by hepatocytes. Furthermore, the metabolism of fructose is less regulated than that of glucose because fructolysis does not involve PFK-1, and this results in the continuous production of pyruvate (the end product of glycolysis). Consequently, in a state of energy surplus, fructose is largely converted to fatty acids and finally to triglycerides [36,37].

**Figure 5:**
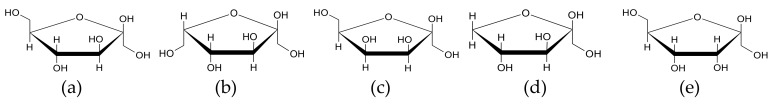
Furanose forms of (**a**) D-fructose, (**b**) L-sorbose, (**c**) D-tagatose, (**d**) D-xylulose, and (**e**) D-allulose.

In the present study, fructose and D-allulose (its epimer) had markedly different metabolic rates in hepatocytes. Fructose is rapidly metabolized with predicted in vivo CL_int_ values of 5.37 and 13.2 mL/min/kg in humans and rats, respectively, whereas D-allulose is highly stable in human or rat hepatocytes, and thus, its CL_int_ cannot be calculated (Table 4). E_h_ values of fructose were also predicted to be low at 0.239 and 0.260 in humans and rats, respectively (Table 4). These results suggest D-allulose is not a substrate of fructokinase which is involved in the first step of fructolysis. Raushel and Cleland reported that the substrate specificity of fructokinase is related to the presence of a furanose ring [38], which is found in β-D-fructofuranose (D-fructose, Figure 5a), α-L-sorbofuranose (L-sorbose, Figure 5b), β-D-tagatofuranose (D-tagatose, Figure 5c) and β-D-xylulofuranose (D-xylulose, Figure 5d), which also indicates a terminal –CH_2_OH group is not required for substrate specificity. On the other hand, β-D-psicofuranose (D-allulose, Figure 5e) was not found to be a substrate for fructokinase, which suggests fructokinase requires a hydroxyl group at carbon 3 trans to the –CH_2_OH group at carbon 2 [38] (Figure 5).

### 3.3. In Vivo Systemic Pharmacokinetic Study in Rats

**Figure 6:**
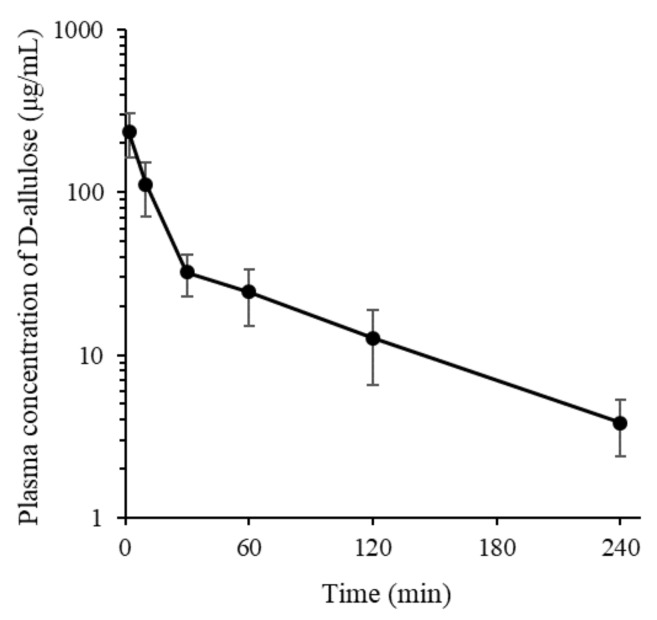
Plasma concentration-time profile of D-allulose after its intravenous administration at 100 mg/kg to rats (*n* = 5). Results are presented as means and error bars represent standard deviation.

Mean arterial plasma concentration–time profiles of D-allulose after intravenous administration at 100 mg/kg to Sprague-Dawley rats (*n* = 5) are shown in Figure 6, and pharmacokinetic parameters determined by noncompartmental analysis are presented in Table 5. Although blood levels of D-allulose after the intravenous administration have only been previously reported at a limited number of time points [18], we report the entire plasma concentration profile of D-allulose and major pharmacokinetic parameters (t_1/2_, CL, and V_ss_). D-allulose concentrations were measurable for up to 240 min after injection and its concentration range in plasma was comparable to that reported in a previous study [18]. D-allulose was rapidly eliminated from plasma with a mean half-life and a total body clearance of 72.2 min and 15.8 mL/min/kg, respectively. Considering the observed stability of D-allulose in rat hepatocytes, this rapid elimination from the systemic circulation was probably due to elimination via the renal route, which concurs with previous reports [18,19].

As mentioned above, excessive sugar consumption may lead to metabolic disorders such as obesity and type 2 diabetes mellitus. Therefore, restrictions on sugar intake and the use of sugar substitutes have been recommended. Non-caloric high-intensity sweeteners such as saccharin, aspartame, acesulfame-k, neotame, sucralose, and advantame, which have 200–20,000 times the sweetness intensity of sucrose have been commonly used as sugar substitute. However, they have been known to have unfavorable reward effects resulting in body weight gain, greater caloric consumption, and glucose intolerance [39,40]. Sugar alcohols (e.g., sorbitol, xylitol, and erythritol) are another type of sugar substitute, and are also commonly referred to as nutritive sweeteners because they have caloric values. In general, sugar alcohols have 30–100% of sweetness of sugar, and most have slightly lower caloric values than sugar, which limits their benefits as sugar substitutes [39,40]. Unlike other sugar alcohols, erythritol produces negligible energy and has been approved for zero-calorie labeling in the United States, Europe, and Japan. Recently, rare sugars such as D-tagatose and D-allulose were approved as food additives and are now widely used as sugar substitutes. These rare sugars are monosaccharides with sweetness similar to those as caloric sugars, but have low caloric values [39,40]. It also has been reported that D-allulose does not cause a satiety or reward response which can affect energy intake [41]. The results obtained during the present study confirmed that D-allulose is a promising sugar substitute because it was not metabolized in the hepatocytes. In addition, D-allulose was rapidly eliminated in vivo probably via urinary excretion, which suggests it is unlikely to raise accumulation-related safety issues.

## 4. Conclusions

In the present study, the in vitro stability of D-allulose was investigated in biorelevant media and in the presence of cryopreserved human and rat hepatocytes. In addition, the metabolisms of fructose and erythritol by hepatocytes were compared with that of D-allulose. It is well known that fructose is utilized as a source of energy and extensively metabolized with little regulation in the livers of animals and humans. On the other hand, sugar alcohol erythritol (an approved food additive) has been known to undergo no metabolism and produce no-calorie in vivo, and our findings confirmed this in human and rat hepatocytes. Likewise, the metabolism of D-allulose by hepatocytes was negligible, whereas fructose was rapidly metabolized by human and rat hepatocytes, which was ascribed to the substrate specificity of the fructolytic pathway. Thus, the observed rapid decline in plasma D-allulose concentration in vivo was probably caused by urinary elimination [18,19]. These observations suggest that D-allulose, like erythritol, is stable in vivo and unlikely to produce calorie in liver. Taken together, D-allulose has advantages that make it comparable to erythritol as a sugar substitute, and thus, it could be used as a food additive, especially in patients with metabolic disease requiring caloric sugar intake limitation due to, for example, obesity, metabolic syndrome, or cardiovascular diseases.

## Figures and Tables

**Table 1:** Composition of simulated gastrointestinal fluids.

Composition	SGF	FaSSIF
NaH_2_PO_4_ (mM)	–	29
NaCl (mM)	34	106
Sodium taurocholate (mM)	0.08	3
Lecithin (mM)	0.02	0.75
Pancreatin (mg/mL)	–	10
Pepsin (mg/mL)	3.2	–
pH	1.6	6.5

**Table 2:** Physiological parameters used for scaling CL_int, in vitro_ to CL_int, in vivo_ in humans and rats.

Parameters	Human	Rat
Number of hepatocyte (cells/g liver)	120 × 10^6^	120 × 10^6^
Liver weight (g liver/kg body weight)	25.7	40
Liver blood flow (mL/min/kg)	20.7	55.2

**Table 3:** Optimized electrospray ionization-mass spectrometry (ESI-MS) parameters including multiple reaction monitoring (MRM) conditions.

Analyte	Molecular Mass	MRM Transition	Collision Energy	Cell Accelerator Voltage
D-allulose	180.156	179.0 → 89.0	2	3
Fructose	180.156	179.0 → 89.0	2	3
Glucose	180.156	179.0 → 89.0	2	3
Erythritol	122.120	121.0 → 71.0	10	3
Salicin (IS)	286.280	285.1 → 123.0	18	3

**Table 4:** In vitro and In vivo intrinsic clearance (CL_int, in vitro_ and CL_int, in vivo_), predicted hepatic clearance (CL_h, in vivo_) and extraction ratio (E_h_) (mean ± standard deviation, *n* = 5).

Compound	CL_int, in vitro_ (μL/min/10^6^ cells) (Human/Rat)	CL_int, in vivo_ (mL/min/kg) (Human/Rat)	Predicted CL_h, in vivo_ (mL/min/kg) (Human/Rat)	E_h_ (Human/Rat)
D-allulose	NC ^1^/NC	NC/NC	NC/NC	NC/NC
Fructose	2.46 ± 1.11/3.63 ± 0.60	7.58 ± 3.43/17.4 ± 2.89	5.37 ± 1.79/13.2 ± 1.70	0.260 ± 0.09/0.239 ± 0.03
Erythritol	NC/NC	NC/NC	NC/NC	NC/NC

^1^ Not calculable.

**Table 5:** Summary of pharmacokinetic parameters of D-allulose after its intravenous administration at 100 mg/kg to rats (mean ± standard deviation, *n* = 5).

Parameters	D-allulose
t_1/2_ (min)	72.2 ± 8.6
AUC_last_ (μg min/mL)	6090 ± 1420
AUC_inf_ (μg min/mL)	6720 ± 1590
CL (mL/min/kg)	15.8 ± 5.0
V_ss_ (mL/kg)	1060 ± 429
MRT (min)	66.9 ± 14.0
